# LGBTQ+ transcultural psychiatry – the need for capacity building, specialized psychiatric services and recognizing the specific needs of LGBTIQ+ refugees in the Nordic countries

**DOI:** 10.1192/j.eurpsy.2025.89

**Published:** 2025-08-26

**Authors:** G. Mijaljica

**Affiliations:** 1 DPS Øyane, Haukeland University Hospital, Bergen, Norway; 2 Transcultural Centre Stockholm, Stockholm, Sweden

## Abstract

**Abstract:**

People within LGBTIQ+ are significantly more likely to suffer a mental health disorder and substance use disorder compared to heterosexual persons. Stigma, discrimination, and lack of social support are considered as some of the contributing factors to the higher incidence of psychiatric morbidity in this group. Simultaneously, the group’s rights vary around the world, and example being that for example homosexuality is criminalized in many countries and sometimes punishable by death.

Even in countries where homosexuality and belonging to LGBTIQ+ community is not criminalized or is well accepted in the society; the studies show increased odds of poor mental health in this group. For example, a study conducted in Sweden, where same – sex marriage is legal, comparing suicide risk between heterosexual and same-sex married couples showed that same-sex married men had a was nearly three-fold greater suicide risk in comparison to their heterosexual counterparts. Accessible and evidence – based LGBTIQ+ affirmative psychiatric services are crucial for addressing the mental health care needs of the group. These services can be supported by well-structured and efficient training of mental health care professionals.

**Disclosure of Interest:**

None Declared

